# Foreign Body in the Œsophagus

**Published:** 1856-06

**Authors:** 


### FOREIGN BODY IN THE ŒSOPHAGUS.

The publication of the case of œsophagotomy, or pharyngotomy, in your Journal of the 9th inst., has induced me to send you a brief report of an accident which fell under my own observation a short time since, and where a similar operation might have become necessary, had not the abstracting body descended before the symptoms resulting from its impaction had become very imminent.

On May 2, 1855, I was consulted by Mrs. D., aged 30, who stated that, during the preceding night, in her sleep, she had accidentally swallowed a piece or plate, containing three artificial teeth, corresponding to the outer incisor, the canine, and first bicuspid. The accident appeared to have been occasioned by this piece having been affixed to a faulty tooth, and thus being too easily detached.

Notwithstanding the inconvenience and suffering thus produced, she had walked to my house, in order, if possible, to get the substance removed. It had passed too far down either to be seen or to be felt by the finger. She referred the sensation produced to a point behind the upper part of the sternum. On having recourse to the probang, I manipulated very gently with it, and after a little time, succeeded in passing it down the œsophagus, hoping that it had carried the extraneous body before it into the stomach, especially as she thought herself much relieved. I prescribed for her a little soothing and aperient medicine.

On the following morning she came to me again, stating that she still felt as if there was some obstruction in the part. On having again recourse to the probang, I met with resistance, near the upper part of the œsophagus, but after a short time, it again passed, inducing us to hope that the obstruction was now removed.

On visiting her at her own residence, two days afterwards, she stated that, from her own sensations, she believed the substance had not really descended into the stomach until the following night after she had called upon me the second time, and on the succeeding day it was discharged through the bowels.

On examining the plate, I found the teeth had not been attached to gold, but to an inferior metal, to lessen expense I suppose.

Except a little soreness in swallowing for a few days, she suffered, I believe, no further inconvenience.

I am, &c., JOHN WINDSOR, F. R. C. S.

Senior Surgeon of the Manchester Eye Hospital. 65 Piccadilly, Manchester, February 14, 1856.—*London Medical Times and Gazette.*

